# Impact of insecticide and fungicide residue contact on plum curculio, *Conotrachelus nenuphar* (Herbst), mobility and mortality: implications for pest management

**DOI:** 10.1002/ps.3445

**Published:** 2012-12-04

**Authors:** Tracy C Leskey, Starker E Wright, Julien Saguez, Charles Vincent

**Affiliations:** a USDA-ARS, Appalachian Fruit Research StationKearneysville, WV, USA; b Agriculture and Agri-Food Canada, Horticulture Research and Development CentreSaint-Jean-Sur-Richelieu, QC, Canada

**Keywords:** plum curculio, mobility, reduced-risk insecticide, IPM

## Abstract

**BACKGROUND:**

An evaluation was made of the effects of seven neurotoxic insecticides (esfenvalerate, indoxacarb, clothianidin, thiacloprid, azinphosmethyl, phosmet and imidacloprid), one insect growth regulator (novaluron) and two fungicides (myclobutanin and mancozeb), with water as the control, on the horizontal mobility of plum curculios exposed to dried pesticide residues. Mobility was recorded over a 2 h period using ethological tracking software. Mortality was recorded immediately after horizontal mobility experiments and 24 h later.

**RESULTS:**

Esfenvalerate had the greatest impact on mobility. Immediately after exposure to this compound, plum curculios moved significantly greater distances and for a significantly longer period of time compared with all other compounds. After 24 h, esfenvalerate also led to high mortality rates (>86.0%). Exposure to azinphosmethyl and phosmet also led to high rates of mortality, although the impact on mobility was less pronounced. Exposure to indoxacarb, thiacloprid, imidacloprid, novaluron, myclobutanin and mancozeb had no impact on mobility and resulted in little to no mortality. Clothianidin affected mobility after a 2 h exposure period, and high mortality (60%) was recorded after 24 h.

**CONCLUSIONS:**

The results indicate that, in the context of a treated orchard, plum curculios exposed to dried pesticide residues may be capable of foraging before succumbing to toxicant exposure, while exposure to pesticides such as esfenvalerate may rapidly incapacitate adult plum curculios.

## INTRODUCTION

The plum curculio, *Conotrachelus nenuphar* Herbst (Curculionidae), is an extremely destructive key pest of stone and pome fruit in commercial orchards in eastern North America.^1^–^3^ As damage from plum curculio can easily exceed 85% if mitigating management procedures are not taken,^4^,^5^ this insect is considered to be a major obstacle to ecological and sustainable pest management programs in tree fruit orchards. For the past 40 years, conventional fruit growers have relied essentially on broad-spectrum insecticides, in particular the organophosphates, to provide commercially acceptable pest control. This typically requires three whole-orchard insecticide treatments to manage plum curculio adults in northeastern apple orchards.^6^,^7^ In all cases, growers are forced to apply insecticides to their entire orchard plots, because plum curculio adults move into^8^ and disperse throughout commercial plots in response to broadly available stimuli and resources.^3^

In the United States, because the use of organophosphates is being severely curtailed and may be eliminated altogether as a result of the 1996 Food Quality Protection Act, growers tend to reduce the amount of organophosphates applied and to replace them with newer insecticides that are often more costly^9^ and have different modes of action, such as oviposition deterrence, antifeedant and repellency effects, or require feeding on treated plant tissue as a mode of toxicant exposure.^10^ The application of these newer insecticides in tree fruit orchards could lead to differences in results obtained from trap-based monitoring procedures,^11^,^12^ for a perimeter row spray management strategy^13^,^14^ or for the trap tree-based management strategy.^15^ Originally, these procedures were developed on the basis of programs using organophosphates such as azinphosmethyl, which has higher acute toxicity and shorter lethal exposure times against adults compared with other newer insecticides,^10^ and also has demonstrated curative activity against eggs and larvae in fruit such as tart cherry.^16^

Plum curculio adults exhibit limited flight at 20 °C,^17^ and consequently much of their movement in and near orchards is achieved by walking.^8^ While foraging for food or oviposition resources in commercial orchards,^8^,^13^ adults likely contact dried residues of applied pesticides, notably on treated trees, groundcover or on pyramidal traps deployed by growers for better timing of insecticide treatments.^3^ However, plum curculio adults are prone to thanatosis, a behavior that involves deliberate dropping to the ground when they perceive a threat,^18^ potentially leading to increased contact, as adults must repeatedly walk up treated surfaces of tree trunks or traps.

In spite of considerable research efforts to design and implement a trapping system for accurate timing of insecticide applications against plum curculio adults, no efficient and reliable trap-based monitoring techniques are available for use in commercial orchards.^12^ Although pyramidal traps are the most frequently deployed trap type used to evaluate adult activity in the field, captures in baited and unbaited pyramidal traps have poor predictive value to determine the risk of damage or the necessity of treatment to prevent damage. Among the factors identified as contributing to unpredictable captures by pyramidal traps,^12^,^19^ the presence of pesticide residues has been overlooked. Furthermore, reduced input strategies such as perimeter row sprays^13^,^14^ and baited trap trees used for both monitoring^20^ and for management^21^ were originally designed using organophosphates.^21^

In order to understand how incidental contact with newer insecticides and cornerstone materials such as organophophates, as well as commonly encountered fungicides, influences trap-based monitoring procedures and reduced input management strategies, plum curculio was utilized as a model organism for mobility bioassays conducted in the laboratory. To quantify the immediate effects of incidental contact with selected pesticide residues on plum curculio behavior, ethological tracking software was used to assess horizontal mobility in no-choice glass arenas treated with pesticides. Mortality rates were also determined. This baseline knowledge will enable better prediction of the immediate effects of insecticides on mobility of plum curculio adults and how this could influence the accuracy of monitoring and the effectiveness of management tactics.

## MATERIALS AND METHODS

### Plum curculios

Adult multivoltine plum curculios were reared in the laboratory.^22^ Immediately after emergence, adults were held in groups of 100 in an environmental chamber for ∼2 weeks at 25 °C and 14:10 h L:D. Adults were then sexed^23^ and returned to the chamber in single-sex groups of approximately 20 individuals held in wax-coated cups (473 mL) with a clear plastic lid and provided with a source of water (wetted cotton dental wick) and green thinning apple as a food source. Adults were starved for 24 h prior to testing to standardize condition.

### Pesticide bioassay

The bioassay was developed on the basis of the underlying assumption that the primary threat posed by plum curculios is from invading adults leaving non-orchard overwintering sites. This movement pattern increases the likelihood that most immigrating individuals will not encounter newly applied, wet pesticide residues, but instead dry residues on the crop. The bioassay consists of measuring insecticide effects on adults after a prescribed exposure period to a dry residue of a pesticide applied to a glass petri dish, hereafter referred to as the arena. This procedure was based on guidelines published by the International Organization of Biological Control^24^ and used to evaluate the effects of thiacloprid on parasitoids.^25^,^26^

Ten pesticides commonly used in orchards were selected to evaluate their effects on adult plum curculios (Table 1). Seven insecticides were assayed: (1) esfenvalerate (Asana® XL 0.66 EC; Dupont™, Wilmington, DE); (2) indoxacarb (Avaunt® 30WDG; Dupont™, Wilmington, DE); (3) clothianidin (Belay® 2.13 SC; Valent® USA Corporation, Walnut Creek, CA); (4) thiacloprid (Calypso® 4 F; Bayer CropScience, Research Triangle Park, NC); (5) azinphosmethyl (Guthion® 50W; Bayer CropScience, Research Triangle Park, NC); (6) phosmet (Imidan® 70 WSB; Gowan Company, Yuma, AZ); (7) imidacloprid (Provado® 1.6 F; Bayer CropScience, Research Triangle Park, NC). One insect growth regulator, novaluron (Rimon® 0.83 EC; Makhteshim Chemical Woks Ltd, Beer Shiva, Israel) was also assayed, and two fungicides, (1) myclobutanil (Nova™ 40 W; Dow AgroSciences™, Indianapolis, IN) and (2) mancozeb (Penncozeb® 75 DF; United Phosphorous Inc., King of Prussia, PA), were evaluated. Water was used as a control.

**Table 1:** Pesticides used to treat arenas

Active ingredient	Class of chemistry^a^	Trial	Recommended field rate (380 L^−1^)	Selected treatment rate (380 L^−1^)	Formulated material (L^−1^)	Active ingredient (g L^−1^)	Active ingredient density (µg cm^−2^)
Esfenvalerate	P	1, 2	6–170 mL	118 mL	0.311 mL	0.025	0.071
Indoxacarb	Ox	1	35–42 g	42 g	0.111 g	0.033	0.093
Indoxacarb	Ox	2	142–170 g	170 g	0.447 g	0.134	0.365
Clothianidin	N	2	177–355 mL	177 mL	0.466 mL	0.119	0.299
Thiacloprid	N	2	30–60 mL	44 mL	0.116 mL	0.056	0.188
Azinphosmethyl	O	1	225–285 g	225 g	0.592 g	0.296	0.816
Phosmet	O	1, 2	454–595 g	510 g	1.342 g	0.939	2.552
Myclobutanil	T	1	35–57 g	42 g	0.111 g	0.044	0.122
Mancozeb	C	1	454 g	454 g	1.195 g	0.896	2.449
Imidacloprid	N	2	30–59 mL	44 mL	0.116 mL	0.022	0.086
Novaluron	IGR	2	591–1479 mL	1479 mL	3.892 mL	0.387	0.990
Water (control)		1, 2	—	—	—	—	0.0

a C: carbamate; IGR: insect growth regulator; N: neonicotinoid; O: organophosphate; Ox: oxadiazine; P: pyrethoid; T: triazole.

Two trials were conducted. In trial 1, selection of pesticides included those that are regularly used to control plum curculio in apple agroecosystems – azinphosmethyl and phosmet (organophosphates), indoxacarb (oxadiazine) and esfenvalerate (pyrethroid), and two commonly used fungicides – mancozeb (ethylene-bis-dithiocarbamates) and myclobutanin (sterol inhibitor), with water as the control. In trial 2, pesticide treatments again included phosmet, esfenvalerate and indoxacarb, as well as insecticides targeting other key pests in apple agroecosystems, including the neonicotinoid insecticides thiacloprid, clothianidin and imidacloprid and the insect growth regulator novaluron, with water once again as the control. Each evaluated compound was formulated on the basis of standard field rates^27^ (Table 1). In trial 1, all rates were based on typical rates selected by growers protecting large standard trees at dilute application rates (2800–3750 L ha^−1^). In trial 2, rates of three materials (clothianidin, indoxacarb and novaluron) were based on current field use patterns, assuming use of concentrated application rates (700–1000 L ha^−1^). The effects of these concentrated materials were compared with those of four dilute compounds (esfenvalerate, phosmet, imidacloprid and thiacloprid).

Arenas consisted of 100 × 15 mm (diameter × height) glass petri dishes covered with plastic lids. Pesticides were mixed with water alone as a carrier. Finished sprays were atomized onto all the interior surfaces of the arena at a volume equal to the delivery rate per unit area (approximately 505 µL per arena) encountered in commercial orchards when using properly calibrated airblast spray equipment. Pesticide residues were allowed to dry completely for 18 h in a fume hood prior to testing. Water was used as a control.

### Horizontal mobility experiments

The effect of dried pesticide residue on horizontal mobility of adult plum curculios was assessed under no-choice conditions over a 2 h exposure period. Each pesticide residue was assessed using a total of 30 adult plum curculios (15 males and 15 females), with a single plum curculio introduced into each arena. The mobility of plum curculio was recorded for 10 min immediately after introduction to the arena and 1 and 2 h later. Between each recording period, plum curculios were maintained in darkness in their respective arenas. Five plum curculios were recorded simultaneously.

To aid in detection of plum curculios in the arenas and to limit glare, trials were conducted in a darkened room, and arenas were backlit using a fluorescent Canon video visualizer stand (model RE-350; Canon, Inc., Japan). Images were captured using a Canon digital video recorder (12× zoom, 5.4–65 mm, 1:1.8) suspended directly above the array of test arenas. Movement tracks were captured live using Noldus EthoVision® software v.3.1.16 (Noldus Information Technologies, The Netherlands), with a capture rate of six samples per second. To separate plum curculios from the background, gray scaling was employed as the detection method, wherein backlit plum curculios always appear darker than the arena base. Noise thresholds were updated manually before each horizontal mobility experiment. Plum curculios were released at the center of an arena and tracked upon capture of a detection area from 3–100 pixels (the maximum area of a mobile subject was recorded at 85 pixels). If lost during a trial, the software recovered the movement track by interpolating the track between the recapture position and the last known position. The whole surface of each arena, including the lid, was contained in a single acquisition zone.

Three parameters were evaluated, and the mean of each was calculated: total distance moved (cm), total duration of movement (s) and velocity (cm s^−1^). To eliminate artificial accumulation of distance moved caused by shifting of the recorded center of the subject plum curculio's mass (cursor fluctuation), an input filter was added on the basis of the subject insect moving a minimum of 0.5 cm. For duration of movement, a subject insect was considered to commence movement upon reaching a velocity of 0.5 cm s^−1^, and was recorded as no longer moving when velocity dropped below 0.1 cm s^−1^.

Accuracy of the input filter for total distance moved and the parameter properties for total duration of movement was validated by correlating manually recorded movement with video capture outputs. Another parameter, maximum velocity (cm s^−1^), was used to separate erroneous track recordings. Data were subsequently analyzed using the GLM procedure^28^ to construct analysis of variance (ANOVA) tables. The models included sex, treatment and the interaction term (sex*treatment) as class variables. Dependent variable data were transformed if homogeneity of variance assumptions were not met according to Levene's test.

### Mortality

Mortality was assessed just after completion of horizontal mobility trials (i.e., after the 2 h period of exposure to dried pesticide residues). Adults were confined individually in plastic containers containing a piece of wetted cotton dental wick and a green thinning apple. Mortality was also checked 24 h later. Percentage mortality included moribund (adults incapacitated and incapable of directed movement) and dead plum curculios.

## RESULTS

### Horizontal mobility

#### Trial 1

The effects of sex and the interaction term (sex*treatment) were not significant immediately after introduction and at 1 and 2 h, and were removed from all models.

**Figure 1:**
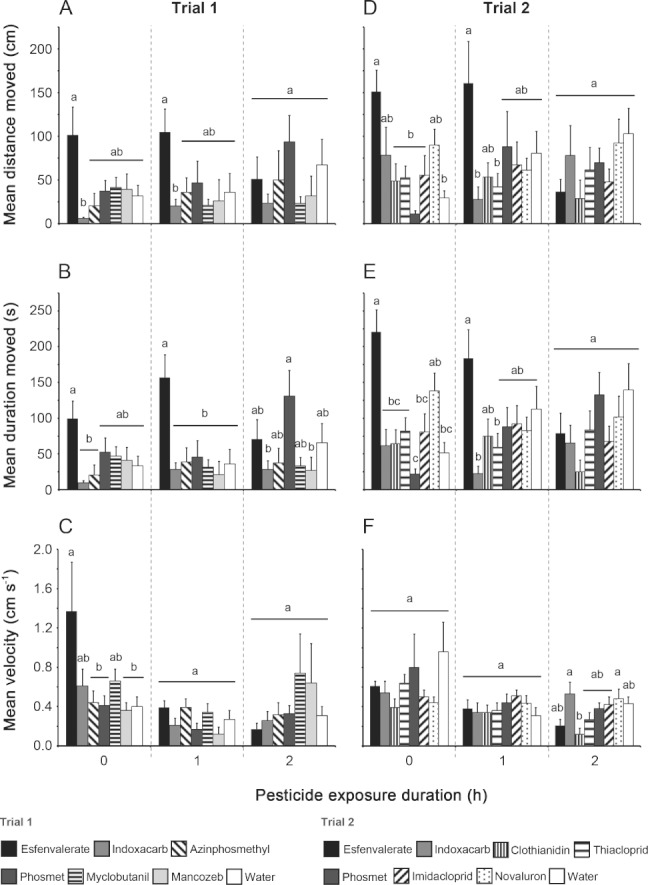
Plum curculio horizontal mobility parameters recorded for 10 min after 0, 1 and 2 h of pesticide or water (control) exposure in trial 1 (A, B, C) and trial 2 (D, E, F). A and D: mean total distance moved (cm); B and E: mean total duration moved (s); C and F: mean velocity (cm s^−1^). Error bars indicate standard errors.

Immediately after introduction into pesticide-treated dishes, there were significant differences in mean distance moved (*F* = 3.18; df = 6, 203; *P* < 0.01), mean duration of movement (*F* = 3.11; df = 6, 203; *P* < 0.01) and mean velocity (*F* = 2.68; df = 6, 203; *P* = 0.02). Plum curculios confined in esfenvalerate-treated arenas moved significantly greater distances (Fig. 1A), spent a significantly greater time period moving compared with those exposed to azinphosmethyl and indoxacarb (Fig. 1B) and moved significantly faster than those introduced into arenas treated with azinphosmethyl, phosmet, mancozeb or water (Fig. 1C).

After 1 h, significant differences were detected in mean distance moved (*F* = 2.19; df = 6, 203; *P* = 0.05) and mean duration of movement (*F* = 6.61; df = 6, 203; *P* < 0.01). Significant differences in mean velocity were not detected among treatments (*F* = 1.84; df = 6, 203; *P* = 0.09). Plum curculios confined in esfenvalerate-treated arenas moved significantly greater distances (Fig. 1A) compared with those confined in arenas treated with indoxacarb, with all other treatments being intermediate. Adults also spent a significantly greater time moving in esfenvalerate-treated arenas compared with arenas treated with all other compounds and the water (control) (Fig. 1B).

After 2 h there were no significant differences in mean distance moved (*F* = 1.09; df = 6, 203; *P* = 0.37) or mean velocity (*F* = 0.85; df = 6, 203; *P* = 0.53) exhibited by adults introduced into pesticide-treated arenas. However, significant differences were detected in mean duration of movement (*F* = 2.58; df = 6, 203; *P* = 0.02). Plum curculios introduced into phosmet-treated arenas spent significantly more time moving compared with those introduced into arenas treated with indoxacarb or mancozeb (Fig. 1B).

#### Trial 2

The effects of sex and the interaction term (sex*treatment) were not significant factors in models for mean distance moved and mean duration of movement for plum curculios immediately after they were introduced into arenas; these factors were removed from the models. However, the effect of treatment was significant for mean distance moved (*F* = 4.72; df = 7, 232; *P* < 0.01) and for mean duration of movement (*F* = 8.37; df = 7, 232; *P* < 0.01). Plum curculios exposed to esfenvalerate moved significantly greater distances than plum curculios in all other treatments except for novaluron and indoxacarb (Fig. 1D), and spent significantly more time moving than plum curculios exposed to all other compounds apart from novaluron (Fig. 1E). The model for mean velocity was significant (*F* = 2.03; df = 15, 224; *P* = 0.01), with only the interaction term (sex*treatment) as a significant factor (*P* < 0.01). Females exposed to water-treated (control) arenas moved significantly faster compared with females exposed to phosmet or imidacloprid.

After 1 h, the model for mean distance moved was significant (*F* = 2.05; df = 15; 224; *P* < 0.01), with the effects of treatment (*P* = 0.03) and the interaction term (*P* < 0.05) being significant. For mean duration of movement, sex and the interaction term were not significant and were removed from the model. However, there was a significant effect of treatment (*F* = 2.49; df = 15, 224; *P* < 0.01). In this case, adults moved significantly greater distances (Fig. 1D) and for significantly longer periods of time (Fig. 1E) when exposed to esfenvalerate compared with thiacloprid or indoxacarb. In addition, females exposed to esfenvalerate moved significantly greater distances compared with females exposed to clothianidin and phosmet (20.27 ± 11.85 cm) and males exposed to indoxacarb. The model for mean velocity was not significant (*F* = 5.96; df = 15, 224; *P* = 0.50), and no differences were observed (Fig. 1F).

After 2 h, the model for mean distance moved was not significant (*F* = 1.62; df = 15, 224; *P* = 0.07), while the model for mean duration of movement was significant (*F* = 2.15; df = 15, 224; *P* < 0.01), with the interaction term as the only significant factor (*P* = 0.02); males exposed to water moved for a significantly longer period of time compared with males exposed to esfenvalerate and females exposed to clothianidin. For mean velocity, sex and the interaction term were not significant and were removed from the model. However, there was a significant effect of treatment (*F* = 2.96; df = 7, 232; *P* < 0.01). Adults exposed to indoxacarb and novaluron exhibited significantly greater mean velocity compared with clothianidin (Fig. 1F).

#### Movement path characteristics

**Figure 2:**
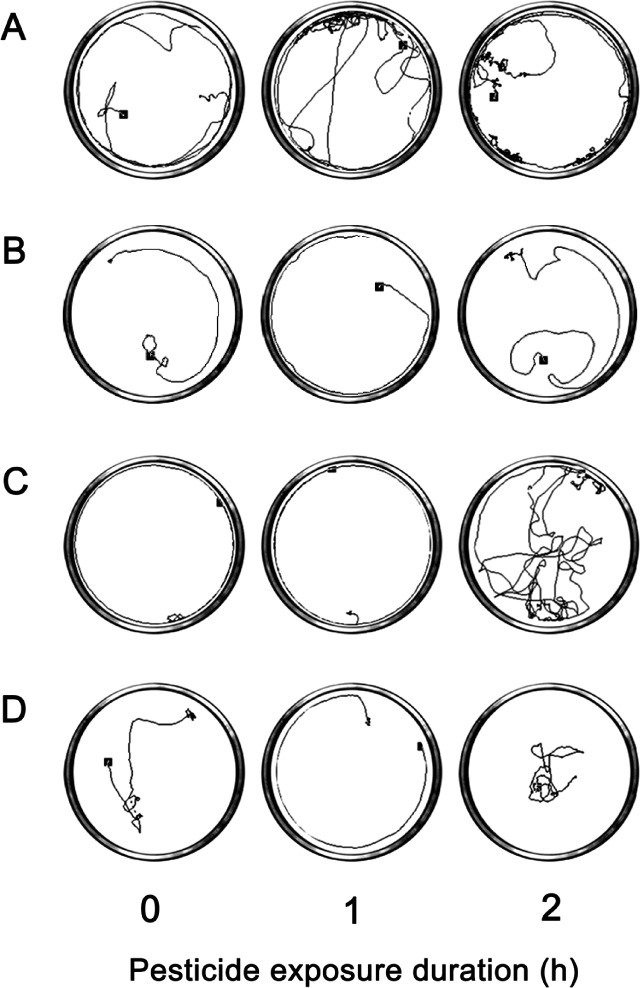
Plum curculio tracks in treated arenas. Representative 10 min tracks recorded after 0, 1 and 2 h of exposure to (A) esfenvalerate, (B) indoxacarb, (C) phosmet and (D) water (control).

The movement path characteristics of adults exposed to esfenvalerate (Fig. 2A) were very different from those of adults exposed to indoxacarb (Fig. 2B) or water (Fig. 2D). After introduction and at 1 h, plum curculios exposed to esfenvalerate moved rapidly around the periphery of the arena, and by 2 h they had begun to succumb. Irregular tracks were often recorded, as plum curculios were no longer able to walk smoothly or continuously. Those exposed to water or indoxacarb showed very little movement. Adults exposed to phosmet (Fig. 2C) also began to succumb after 2 h and also often produced irregular tracks across the surface of the dish, although they generally did not fall over, as was observed in the esfenvalerate treatment.

### Mortality

After 2 h exposure, mortality was greatest for adults exposed to esfenvalerate residues with respectively 83.3 and 76.6% in trial 1 and trial 2 (Table 2). After 24 h, mortality of adults exposed to azinphosmethyl and phosmet had increased to 93.3 and 80.0% respectively in trial 1, and to 46.6% for phosmet in trial 2, while those introduced into the esfenvalerate-treated arenas increased to 86.6 and 93.3% in trials 1 and 2 respectively. For indoxacarb, no mortality based on residue exposure was detected in trial 1, and only 6.7% in trial 2. Fungicides induced no mortality after a 2 h exposure period, and no or negligible mortality after 24 h on adult plum curculios. Among other insecticides evaluated in trial 2, exposure to clothianidin resulted in 60.0% mortality after 24 h, but there was a decline from the 2 h assessment (76.7%), as 16.7% of adults did recover from a moribund state.

**Table 2:** Plum curculio mortality (%) at 0 and 24 h following horizontal mobility experiments, i.e. after a 2 h period of exposure to pesticides or water (control)

Pesticide	Trial	0 h	24 h
Esfenvalerate	1	83.3	86.6
Esfenvalerate	2	76.7	93.3
Indoxacarb	1	0.0	0.0
Indoxacarb	2	0.0	6.7
Clothianidin	2	76.7	60
Thiacloprid	2	0.0	26.7
Azinphosmethyl	1	30.0	93.3
Phosmet	1	20.0	80.0
Phosmet	2	16.7	46.7
Myclobutanil	1	0.0	0.0
Mancozeb	1	0.0	6.6
Imidacloprid	2	0.0	3.3
Novaluron	2	0.0	3.3
Water (control)	1, 2	0.0	0.0

## DISCUSSION

Among the compounds tested, the most immediate impact on mobility was found to be from esfenvalerate, a pyrethroid. Recently, the term ‘locomotive stimulant’ was applied to describe the behavior of Culicidae^29^ contacting pyrethroid-treated surfaces. It appears that esfenvalerate, a type II pyrethroid, serves as such a chemical for plum curculio, as adults immediately moved greater distances and spent more time moving and at greater velocities than when exposed to other chemicals. However, within 2 h, the mean horizontal distance traveled by adults declined by ∼50%. Within orchards, it seems likely that adults contacting residues while foraging within treated canopies would succumb very quickly. Similar patterns of mortality have been observed for Culicidae when they have increased contact with pyrethroid-treated surfaces.^30^ And, although pyrethroids have long been considered to be effective against plum curculio,^27^ they are also considered to be problematic because of their tendency to flare populations of phytophagous mites such as the European red mite, *Panonychus ulmi* (Koch),^31^,^32^ and two-spotted spider mite, *Tetranychus urticae* (Koch),^33^ owing to toxicity to predaceous mites and other natural enemies.^33^,^34^ However, a pyrethroid may be a good fit if used as part of a targeted and spatially limited management technique. Indeed, perimeter row spray treatments^13^,^14^ and trap tree management strategy,^21^ an attract-and-kill strategy based on deploying lures in select perimeter row trees, may benefit from using a pyrethroid because of the fast knockdown of plum curculio and because the insecticide treatment would be limited to either the perimeter row of the orchard or to a few selected perimeter row trees, reducing the potential for disruption of natural enemies. However, pyrethroids degrade quickly under field conditions,^35^ and, because of this, they may need to be applied more frequently, although some newer formulations are thought to be more stable.

No impact of fungicides on mobility of plum curculio was seen. Similarly, fungicides typically applied in greenhouses did not affect adult rove beetles, *Atheta coriara* (Coleoptera: Staphylinidae).^36^ Newer ‘reduced-risk’ compounds are now commonly applied as part of IPM programs in tree fruit. These include neonicotinoids which are also toxic to plum curculio and have shown oviposition deterrence, antifeedant and repellency effects.^10^,^16^ The results demonstrated no measurable effects of two neonicotinoids, thiacloprid and imidacloprid, on horizontal mobility of plum curculio, based on the present 2 h exposure interval. However, clothianidin did ultimately result in declines in overall horizontal mobility after 2 h of exposure, and this did translate into substantial mortality. Mortality of adults was low for thiacloprid and imidacloprid, suggesting that a longer exposure interval may be necessary for any effects on mobility to be detectable. The insect growth regulator “novaluron” was shown to reduce plum curculio larval survivorship following mated female exposure, i.e. vertical transmission following exposure to a treated substrate.^37^ In terms of adult response to dry residue, the present studies revealed no immediate effects on mobility or mortality. Thus, in the context of a treated orchard, it is important to consider whether a compound will have an immediate impact on the foraging ability of plum curculio or whether a longer period of exposure for dilute applications or higher concentrations of toxicant may be required before adults succumb to insecticide exposure.

A serious problem that has plagued the development of trap-based monitoring systems for plum curculio in sprayed orchards is the lack of reliable relationships between trap captures and injury,^11^,^12^ which may be partly due to competition between baited traps and stimuli provided by rapidly growing fruit, leading to declining captures after petal fall.^19^ However, the present results indicate that incidental contact with some insecticides such as esfenvalerate or phosmet may rapidly affect adult plum curculios and impair their normal locomotory behavior. Thus, exposure to dried pesticide residues prior to entering the collection device of a trapping unit may alter trap captures. The trapping mechanism used for plum curculios is quite different to that for lepidopteran tree fruit pests, which are monitored by exploiting male flight patterns; males orient directly to and are captured within pheromone-baited traps, limiting their exposure to pesticide residues prior to alighting on a trap. Although plum curculio movement is temperature dependent and adults fly at temperatures above 20 °C,^17^ adults generally forage in and around host fruit tree canopies by walking,^38^ increasing the time of body contact with insecticide residue. Thus, if it takes 2 h or longer for a plum curculio to reach a trap collection device, i.e., the capture mechanism, it no longer may be able to climb vertically or be captured, contributing to a lack of relationship between captures and injury. A simple way to mitigate this problem is to cover traps during treatments or to remove them from treated blocks prior to pesticide applications. This does not preclude plum curculio exposure to residues present on groundcover if they are walking. However, such an approach would allow for traps to remain unaffected by pesticide residues, thereby reducing the likelihood of spurious pest management decisions based on unreliable trap captures. However, use of insecticides such as indoxacarb that require plum curculio to ingest treated plant tissue in order to achieve optimal toxicity^10,39^ may be more compatible with trap-based monitoring systems used in fruit orchards, as no detectable effects on horizontal mobility were found at dilute or concentrated application rates.

## CONCLUSION

The experiments were designed to expose adults to a single pesticide for a designated period of time under no-choice conditions. However, in the context of a managed orchard, the conditions would be quite different in that foraging adults would be exposed simultaneously to multiple pesticides (e.g. fungicides, herbicides, insecticides and acaricides) applied at varying field rates and of constantly changing toxicity levels based on residue age and abiotic conditions. Thus, measurable effects of incidental contact with pesticide residues on foraging behavior of adult plum curculios will likely become less predictable. However, understanding the impact of key insecticides on foraging behavior should enable growers to make informed decisions regarding treatment options and monitoring strategies.

## References

[1] Racette G, Chouinard G, Vincent C, Hill SB (1992). Ecology and management of the plum curculio, *Conotrachelus nenuphar* (Herbst) (Coleoptera: Curculionidae). Phytoprotection.

[2] Vincent C, Chouinard G, Hill SB (1999). Progress in plum curculio management: a review. Agric Ecosyst Environ.

[3] Leskey TC, Chouinard G, Vincent C, Aluja M, Leskey TC, Vincent C (2009). Monitoring and management of the apple maggot fly and the plum curculio: honoring the legacy of R. J. Prokopy. Biorational Tree Fruit Pest Management.

[4] Vincent C, Roy M (1992). Entomological limits to the implementation of biological programs in Quebec apple orchards. Acta Phytopathol Entomol Hung.

[5] Prokopy RJ, Chandler B, Dynok SA, Piñero JC (2003). Odor-baited trap trees: a new approach to monitoring plum curculio (Coleoptera: Curculionidae). J Econ Entomol.

[6] Prokopy RJ, Mason JL, Christie M, Wright SE (1996). Arthropod and natural enemy abundance under second-level vs. first-level integrated pest management practices in apple orchards: a four year study. Agric Ecosyst Environ.

[7] Reissig WH, Nyrop JP, Straub R (1998). Oviposition model for timing insecticide sprays against plum curculio in New York State. Environ Entomol.

[8] Lafleur G, Hill SB (1987). Spring migration, within-orchard dispersal, and apple-tree preference of plum curculio (Coleoptera: Curculionidae) in southern Quebec. J Econ Entomol.

[9] Shearer PW, Atanassov A, Rucker A (2006). Eliminating organophosphate and carbamate insecticides from New Jersey, USA, peach culture. Acta Hortic.

[10] Wise JC, Coombs AB, Vandervoort C, Hoffmann EJ, Whalon ME (2006). Use of residue profile analysis to identify modes of insecticide activity contributing to control of plum curculio. J Econ Entomol.

[11] Prokopy RJ, Chandler B, Dynok SA, Piñero JC (2003). Odor-baited trap trees: a new approach to monitoring plum curculio (Coleoptera: Curculionidae). J Econ Entomol.

[12] Leskey TC, Wright SE (2004). Monitoring plum curculio, *Conotrachelus nenuphar* (Herbst) (Coleoptera: Curculionidae), populations in apple and peach orchards in the mid-Atlantic. J Econ Entomol.

[13] Chouinard G, Hill SB, Vincent C, Barthakur NN (1992). Border-row sprays against the plum curculio (Coleoptera: Curculionidae) in apple orchards: a behavioral study. J Econ Entomol.

[14] Vincent C, Chouinard G, Bostanian NJ, Morin Y (1997). Peripheral zone treatments for plum curculio management: validation in commercial apple orchards. Entomol Exp Applic.

[15] Leskey TC, Piñero JC, Prokopy RJ (2008). Odor baited trap trees: novel management tool for the plum curculio. J Econ Entomol.

[16] Hoffmann EJ, Vandervoort C, Wise JC (2009). Curative activity of insecticides against plum curculio (Coleoptera: Curculionidae) in tart cherries. J Econ Entomol.

[17] Prokopy RJ, Wirth CB, Leskey TC (1999). Movement of plum curculios toward host trees and traps: flight versus walking. Entomol Exp Applic.

[18] Racette G, Hill SB, Vincent C (1990). Actographs to record the daily activity of the plum curculio (Coleoptera: Curculionidae). J Econ Entomol.

[19] Leskey TC, Wright SE (2004). Influence of host tree proximity on adult plum curculio (Coleoptera: Curculionidae) responses to monitoring traps. Environ Entomol.

[20] Piñero JC, Agnello AM, Tuttle A, Leskey TC, Faubert H, Koehler G (2011). Effectiveness of odor-baited trap trees for plum curculio (Coleoptera: Curculionidae) in commercial apple orchards in the Northeast. J Econ Entomol.

[21] Leskey TC, Piñero JC, Prokopy RJ (2008). Odor baited trap trees: novel management tool for the plum curculio. J Econ Entomol.

[22] Amis A, Snow JJ, Singh P, Moore RF (1985). Conotrachelus nenuphar. Handbook of Insect Rearing.

[23] Thomson RR (1932). Sex differentiation of adults of *Conotrachelus nenuphar*. J Econ Entomol.

[24] Candolfi MP, Blümel S, Forster R, Bakker FM, Grimm C, Hassan SA (2000). Guidelines to evaluate side-effects of plant protection products to non-target arthropods.

[25] Schuld M, Schmuck R (2000). Effects of thiacloprid, a new chloronicotinyl insecticide, on the egg parasitoid *Trichogramma cacoeciae*. Ecotoxicology.

[26] Grimm C, Candolfi MP, Fisch R (2002). A comparison of rate–response toxicity tests with *Aphidius rhopalosiphi* (Hymenoptera: Aphidiidae) using glass, leaves, and whole plants as substrates. Chemosphere.

[27] Pfeiffer DG http://pubs.ext.vt.edu/ANR/ANR-4/ANR-4_pdf.pdf.

[28] (2003). SAS Software: Version 8.2.

[29] Cooperband MF, Allan SA (2009). Effects of different pyrethroids on landing behavior of female *Aedes aegypti**Anopheles quadrimaculatus*, and *Culex quinquefasciatus* mosquitoes (Diptera: Culicidae). J Med Entomol.

[30] Siegert PY, Walker E, Miller JR (2009). Differential responses of *Anopheles gambiae* (Diptera: Culicidae) modulate mortality caused by pyrethroid-treated bednets. J Econ Entomol.

[31] Hall FR (1979). Effects of synthetic pyrethroids on major insect and mite pests of apple. J Econ Entomol.

[32] Hull LA, Beers EH, Meagher RL (1985). Impact of selective use of synthetic pyrethroid fenvalerate on apple pests and natural enemies in large-orchard trials. J Econ Entomol.

[33] Hull LA, Starner VA (1983). Impact of four synthetic pyrethroids on major natural enemies and pests of apple in Pennsylvania. J Econ Entomol.

[34] Cloyd RA, Timmons NR, Goebel JM, Kemp KE (2009). Effect of pesticides on adult rove beetle *Atheta coriaria* (Coleoptera: Staphylinidae) survival in growing medium. J Econ Entomol.

[35] Wise JC, Kim K, Hoffmann EJ, Vandervoort C, Gökçe A, Whalon ME (2007). Novel life stage targets against plum curculio, *Conotrachelus nenuphar* (Herbst), in apple integrated pest management. Pest Manag Sci.

[36] Chouinard G, Hill SB, Vincent C (1994). Spatial distribution and movement of plum curculio adults within caged apple trees. Entomol Exp Appl.

[37] Wise J, Whalon M, Ishaaya I, Horowitz AR (2009). A systems approach to IPM integration, ecological assessment and resistance management in tree fruit orchards. Biorational Control of Arthropod Pests.

